# Financial toxicity and exercise adherence in stroke patients: a parallel mediation model of perceived social support and self-efficacy

**DOI:** 10.3389/fpubh.2026.1814437

**Published:** 2026-05-20

**Authors:** Xin Luo, Ying Peng, Qinghua Zhao

**Affiliations:** 1 Department of Nursing, The First Affiliated Hospital of Chongqing Medical University, Chongqing, China; 2 Center of Nursing Research, The First Affiliated Hospital of Chongqing Medical University, Chongqing, China

**Keywords:** exercise adherence, financial toxicity, mediating effect, perceived social support, self-efficacy, stroke

## Abstract

**Objective:**

To analyze the relationship between financial toxicity and exercise adherence among stroke patients and to test the parallel mediating effects of perceived social support and self-efficacy.

**Methods:**

Using convenience sampling, 362 stroke patients were surveyed from September 2024 to July 2025. The survey instruments included Basic Information Questionnaire, Comprehensive Scores for Financial Toxicity Based on the Patient-Reported Outcome Measures, Stroke Self-Efficacy Questionnaire, Perceived Social Support Scale, and Questionnaire of Exercise Adherence. Pearson correlation analysis, structural equation modeling, and Bootstrap methods were applied to examine the connections among stroke patients’ financial toxicity, self-efficacy, perceived social support, and exercise adherence.

**Results:**

The correlations between financial toxicity, self-efficacy, perceived social support, and exercise adherence were statistically significant (*p* < 0.01). The structural equation model fit well, and the main paths were statistically significant (*p* < 0.05). Bootstrap results showed that the direct effect of financial toxicity on exercise adherence was 0.279 (95% CI [0.199, 0.359]), the indirect effect of self-efficacy was 0.152 (95% CI [0.103, 0.208]), and the indirect effect of perceived social support was 0.063 (95% CI [0.035, 0.094]).

**Conclusion:**

Financial toxicity is significantly associated with exercise adherence among stroke patients. An indirect association was also observed between financial toxicity and exercise adherence via self-efficacy and perceived social support. This suggests that in clinical practice, in addition to addressing financial toxicity, it is also important to enhance patients’ self-efficacy and strengthen social support to promote exercise adherence.

## 1 Introduction

Stroke is the third most common cause of death globally and the fourth most common cause of disability-adjusted life years, representing a substantial disease burden (1). Globally, the total direct medical costs and productivity losses caused by stroke exceed US$891 billion annually, accounting for 0.66% of the global GDP (2). In China, the total annual economic burden caused by stroke is about 952.2 billion yuan, imposing a heavy pressure on both society and families (3). Financial toxicity is defined as the objective financial burden and subjective financial distress caused by out-of-pocket medical expenses during disease treatment (4). Financial toxicity was first mainly applied to cancer patients and was later gradually extended to chronic diseases such as cardiovascular diseases (5). Previous research has found that stroke patients are widely affected by financial toxicity (6–8). In addition, many studies have shown that financial toxicity has negative effects on patients’ treatment decisions and health-related behaviors (9–12). Three studies indicated that financial toxicity leads patients to delay or abandon treatment (9, 10, 12). One study suggested that financial toxicity is associated with greater anxiety and depressive symptoms (12). One study found that financial toxicity is associated with multiple adverse outcomes, including a decline in health-related quality of life and involuntary lifestyle changes due to financial constraints (11). This suggests that we should consider the potential health impacts of financial toxicity on stroke patients.

Exercise after stroke is helpful to promote functional recovery and improve activities of daily living (13). However, during the rehabilitation process, exercise adherence among stroke patients still varies. Previous studies have found that both the economic conditions and psychological status of stroke patients may influence their exercise adherence (14, 15). From an economic perspective, limited financial resources may restrict access to rehabilitation services, exercise guidance, and supportive equipment (16). From a psychological perspective, worry and reduced confidence in managing rehabilitation tasks may weaken motivation and persistence, especially when recovery is slow or symptoms fluctuate (17). This suggests that there is a connection between financial toxicity and exercise adherence. In this context, financial toxicity reflects the financial burden and psychological stress related to disease and treatment. It may reduce patients’ ability and willingness to maintain regular exercise, thereby undermining rehabilitation behaviors and treatment outcomes.

Self-efficacy refers to an individual’s belief in their capacity to orchestrate and execute particular actions to attain anticipated results (18). In the context of stroke rehabilitation, this belief is closely related to whether patients can initiate exercise, maintain regular practice, and persist when they experience fatigue, setbacks, or slow functional improvement. Studies have indicated that more severe financial toxicity correlates with lower patient self-efficacy (12). When patients experience financial strain, they may face ongoing concerns about treatment costs and family burden. These concerns can reduce confidence in completing rehabilitation tasks. During rehabilitation, self-efficacy is considered to be an important psychological factor affecting patients’ persistence in exercise (19). Relevant research has indicated that self-efficacy has an impact on exercise adherence among stroke patients (20). In summary, financial toxicity may indirectly influence exercise adherence by weakening patients’ self-efficacy. Therefore, this study aims to verify the mediating function of self-efficacy in the link between financial toxicity and exercise adherence among stroke patients.

Perceived social support (PSS) refers to an individual’s subjective awareness of the accessibility of assistance from family, friends, and other sources (21). Research has demonstrated that effective social support can help reduce patients’ economic pressure during disease treatment (22). For example, support from family and social networks may provide direct financial help, assist with medical decision-making, or share caregiving responsibilities. Such support may reduce the sense of being overwhelmed by medical expenses. Evidence also indicates that perceived social support can promote patients’ adherence to exercise (23). Research has found that PSS can relieve patients’ economic burden and psychological distress, thereby improving their exercise adherence (24). It follows that financial toxicity may indirectly affect exercise adherence by influencing patients’ perceived social support. Therefore, this study will explore the mediating role of PSS in the association between stroke patients’ financial toxicity and exercise adherence.

Stress and coping theory was proposed by Lazarus (25). This theory conceptualizes stress as a product of the interaction between the individual and the environment. Stress occurs when a person appraises internal or external demands as exceeding their coping capacity and available coping resources. Whether a stressor leads to stress depends largely on two key psychological processes: cognitive appraisal and coping. Cognitive appraisal refers to the cognitive process by which an individual perceives whether a situation has an impact on them. It includes identifying and reflecting on sources of stress (primary appraisal) and evaluating one’s own coping abilities (secondary appraisal). Individuals first use primary appraisal to judge whether an event pertains to their own interests. If the primary appraisal indicates stress, a secondary appraisal will be conducted. During secondary appraisal, individuals evaluate their coping ability and available coping resources, and then adopt corresponding coping strategies. In this study, financial toxicity in stroke patients is considered a stressor. The patients then evaluate their coping ability and available coping resources. Patients’ self-efficacy and perceived social support correspond to coping abilities and coping resources. Patients’ assessment of their self-efficacy and perceived social support may influence their coping behaviors, and then lead to different levels of rehabilitation exercise adherence.

**Figure 1:**
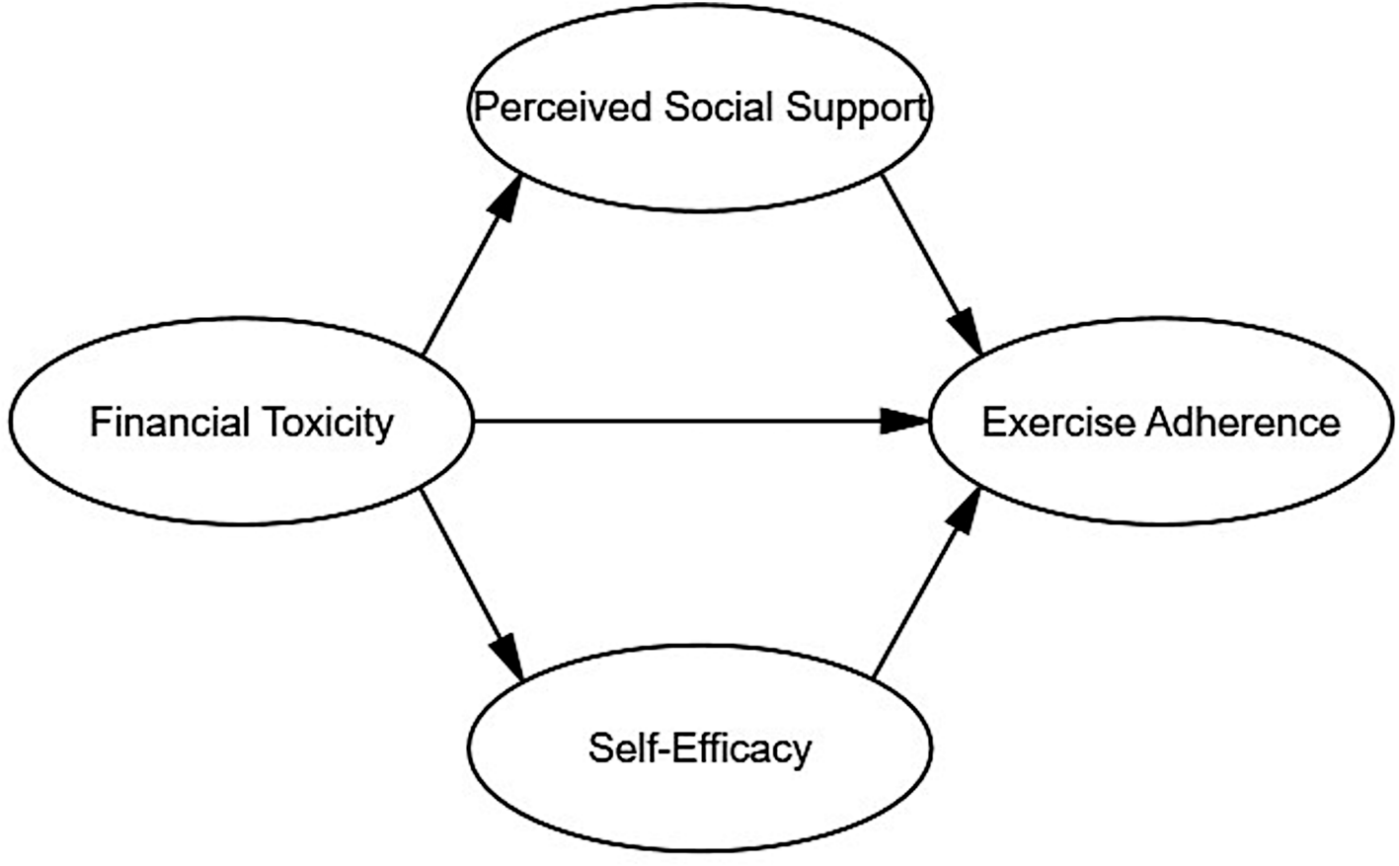
The hypothetical model.

Therefore, based on stress and coping theory, self-efficacy and perceived social support may mediate the association between financial toxicity and exercise adherence among stroke patients. Based on the literature review and theoretical framework described above, the hypothesized model is presented in Figure 1. The structural equation model (SEM) will be used to examine the parallel mediating roles of self-efficacy and perceived social support in the association between stroke patients’ financial toxicity and exercise adherence.

## 2 Methods

### 2.1 Study design and participants

This study used convenience sampling. From September 2024 to July 2025, a cross-sectional survey was carried out in a tertiary hospital in Chongqing, China. Eligible participants were recruited and invited to complete a questionnaire survey. A total of 362 stroke patients participated in the study.

The inclusion criteria were as follows: (1) clinically diagnosed with ischemic stroke or intracerebral hemorrhage by a physician according to the medical records, with stroke-related functional impairment requiring rehabilitation management; (2) age ≥ 18 years; (3) muscle strength grade ≤ 4, assessed using Manual Muscle Testing; (4) clear consciousness and able to communicate effectively; (5) informed about the study and willing to participate. The exclusion criteria were: (1) transient ischemic attack, because it usually does not involve persistent dysfunction requiring long-term rehabilitation exercise; (2) cognitive or language impairment that prevented accurate understanding or responses to the questionnaire; (3) being in the terminal stage of life; (4) incomplete data collection.

The study protocol was approved by the Medical Research Ethics Committee of the First Affiliated Hospital of Chongqing Medical University (approval number: 2024-265-01).

### 2.2 Sample size calculation

Monte Carlo power analysis for indirect effects was used to estimate the sample size (26). The calculation was performed using an online tool.^1^ A preliminary survey was conducted on 55 eligible stroke patients. The correlation coefficients among financial toxicity, self-efficacy, perceived social support, and exercise adherence: *r*_1_ = 0.579, *r*_2_ = 0.587, *r*_3_ = 0.677, *r*_4_ = 0.726, *r*_5_ = 0.807, and *r*_6_ = 0.695. Using these correlation coefficients as input parameters, with *α* = 0.05 and power (1 − *β*) = 0.90, the smallest sample size required for this study was calculated as 228.

### 2.3 Research instruments

#### 2.3.1 Basic information questionnaire

The questionnaire was self-designed. It covered age, gender, education level, residence, medical insurance status, number of comorbid chronic diseases, and activity of daily living (evaluated using the Barthel Index).

#### 2.3.2 Comprehensive scores for financial toxicity based on the patient-reported outcome measures (COST-PROM)

De Souza and associates developed the COST-PROM to measure patients’ financial toxicity (27). Yu et al. (28) localized the Chinese version of the scale. This scale consists of 11 items, including two dimensions: positive wealth status and negative psychosocial responses (29). Each item on the scale is scored from 0 to 4, with the cumulative score range of 0–44. Lower scores suggest greater financial toxicity. According to the grading criteria used in previous studies, a total score of 0 indicates severe financial toxicity; a score <14 indicates moderate financial toxicity; a score <26 indicates mild financial toxicity; and a score ≥26 indicates no financial toxicity (30). The COST-PROM showed good internal consistency reliability in this investigation (Cronbach’s *α* = 0.919).

#### 2.3.3 Stroke self-efficacy questionnaire (SSEQ)

Jones et al. (31) developed the SSEQ to assess self-efficacy during functional training in stroke patients. Li et al. (32) localized the Chinese version of the scale. It includes 11 items, covering two dimensions: efficacy in activities of daily living and self-management efficacy. Each item is scored from 0 to 10. 0–10 indicates a range from no confidence to complete confidence. The overall score ranges from 0 to 110. Higher scores indicate higher levels of self-efficacy. In this investigation, the SSEQ’s internal consistency reliability was good (Cronbach’s *α* = 0.899).

#### 2.3.4 Perceived social support scale (PSSS)

Zimet et al. (33) developed the PSSS. The Chinese version of the scale was adapted by Jiang (34). The scale consists of 12 items, comprising three dimensions: family support, friend support, and support from others. Each item is rated from 1 to 7, ranging from “strongly disagree” to “strongly agree,” with the cumulative score range of 12–84. A higher score reflects a greater level of PSS. In this investigation, the PSSS showed good internal consistency (Cronbach’s *α* = 0.845).

#### 2.3.5 Questionnaire of exercise adherence (EAQ)

The EAQ was developed by Lin et al. (35). It was used to assess the rehabilitation exercise adherence of stroke patients. The scale contains 14 items and includes three dimensions: physical participation in exercise, monitoring of exercise effects, and actively seeking exercise advice. Each item is scored from 1 to 4, ranging from “not at all able to do” to “completely able to do”, with an overall score range of 14–56. Higher scores reflect superior exercise adherence. In this investigation, the EAQ demonstrated good internal consistency reliability (Cronbach’s *α* = 0.911).

### 2.4 Data collection

Prior to the questionnaire survey, all data collectors received standardized training to ensure a consistent understanding of the survey procedures and uniform administration of the questionnaire. During data collection, two trained investigators distributed the questionnaires to participants on site and collected them immediately after completion. Before administering the survey, the investigators explained the purpose and significance of the study to participants. After obtaining the patients’ informed consent, the investigators provided relevant explanations regarding the survey content using unified guidance statements. After data collection, the questionnaires were checked promptly for completeness. If any items were missing, the investigators asked participants to complete them in a timely manner. A total of 370 questionnaires were distributed, and 362 were valid, yielding a valid response rate of 97.8%.

### 2.5 Statistical analysis

Data were entered into Excel and then imported into statistical software for analysis. Continuous variables were described using the mean and standard deviation (SD). Categorical variables were described using frequencies (*n*) and percentages (%). The correlation between the main variables (financial toxicity, self-efficacy, perceived social support, and exercise adherence) was analyzed using Pearson correlation analysis. AMOS 29.0 was used to construct the SEM. Age, gender, and activity of daily living were included in the model as covariates. The fit of the model was evaluated using multiple indices. These included the Normed Chi Square Index (*χ*^2^/df), Root Mean Square Error of Approximation (RMSEA), Goodness of Fit Index (GFI), Comparative Fit Index (CFI), Tucker and Lewis Index (TLI), and Incremental Fit Index (IFI). When *χ*^2^/df < 3, RMSEA < 0.08, and GFI, CFI, TLI, IFI ≥ 0.90, the model fits well (36). The mediation effect was examined through the Bootstrap method. Statistical significance was indicated when the 95% confidence interval (CI) did not include 0.

## 3 Results

### 3.1 Characteristics of participants

A total of 362 patients with stroke were included. Their basic characteristics are shown in Table 1. The average age of participants was 63.72 ± 12.22 years. Most participants were aged ≥ 60 years (64.9%), and 67.4% were male. Educational attainment was generally low, with 37.0% reporting primary school education or below. Most participants were covered by medical insurance, mainly employee medical insurance (53.3%) or resident medical insurance (39.0%). Comorbid chronic diseases were common, with 76.5% reporting at least one comorbidity. Regarding activity of daily living, 51.5% had mild dependence, 24.3% had moderate dependence, and 24.3% had severe dependence.

**Table 1:** Characteristics of participants (*N* = 362).

Variables	Categories	*n* (%)
Age	18–44	30 (8.3%)
	45–59	97 (26.8%)
	≥60	235 (64.9%)
Gender	Male	244 (67.4%)
	Female	118 (32.6%)
Education level	Primary school or below	134 (37.0%)
	Middle school	104 (28.7%)
	High school	54 (14.9%)
	College or above	70 (19.3%)
Residence	Urban	269 (74.3%)
	Rural	93 (25.7%)
Medical insurance status	No medical insurance	28 (7.7%)
	Employee medical insurance	193 (53.3%)
	Resident medical insurance	141 (39.0%)
Number of comorbid chronic diseases	0	85 (23.5%)
	1	159 (43.9%)
	2	82 (22.7%)
	≥3	36 (9.9%)
Activity of daily living	Mild dependence	186 (51.5%)
	Moderate dependence	88 (24.3%)
	Severe dependence	88 (24.3%)

### 3.2 Correlation analysis of financial toxicity, self-efficacy, perceived social support, and exercise adherence

The means of financial toxicity, self-efficacy, perceived social support, and exercise adherence were 20.57 ± 7.52, 64.87 ± 16.61, 58.89 ± 9.01, 37.00 ± 7.16, respectively. The findings of the correlation analysis among the four variables are shown in Table 2. Financial toxicity (with higher COST-PROM scores indicating lower levels of financial toxicity) was positively correlated with self-efficacy (*r* = 0.435, *p* < 0.01), perceived social support (*r* = 0.277, p < 0.01), and exercise adherence (*r* = 0.517, p < 0.01). Both self-efficacy (*r* = 0.591, p < 0.01) and perceived social support (*r* = 0.469, p < 0.01) showed significant positive correlations with exercise adherence.

**Table 2:** Correlation analysis of scale scores in patients with stroke (*N* = 362).

Variables	Mean	SD	1	2	3	4
1. Financial toxicity	20.57	7.52	1			
2. Self-efficacy	64.87	16.61	0.435^**^	1		
3. Perceived social support	58.89	9.01	0.277^**^	0.412^**^	1	
4. Exercise adherence	37.00	7.16	0.517^**^	0.591^**^	0.469^**^	1

^**^p < 0.01.

### 3.3 Parallel mediation analysis

This study employed Harman’s single-factor test to evaluate the severity of common method bias. According to the findings, 10 common factors had eigenvalues higher than 1. Less than 40% of the variance, or 28.83%, was explained by the first factor. This suggests that no serious common method bias exists (37).

**Figure 2:**
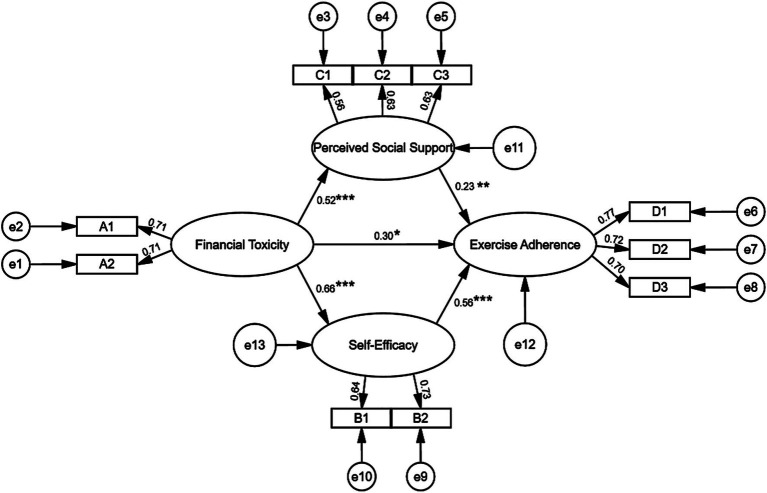
Structural equation model. A1, Positive wealth status; A2, negative psychosocial responses; B1, efficacy in activities of daily living; B2, self-management efficacy; C1, friend support; C2, other support; C3, family support; D1, physical participation in exercise; D2, monitoring of exercise effects; D3, actively seeking exercise advice. ^***^p < 0.001, ^**^p < 0.01, ^*^p < 0.05.

The SEM of parallel mediation is shown in Figure 2. The model fit results indicate: *χ*^2^/df = 2.392, RMSEA = 0.062, GFI = 0.953, CFI = 0.939, TLI = 0.901, IFI = 0.940. All fit indices satisfied the recommended criteria, indicating a good model fit. After controlling for covariates, all main paths in the SEM were statistically significant (*p* < 0.05). Among the covariates, activity of daily living showed statistical significance with self-efficacy and exercise adherence. Age and gender were not statistically significant.

The mediation effect was examined via the Bootstrap method, based on 5,000 resamples. Table 3 displays the findings. The effect size of financial toxicity on exercise adherence was 0.279 (95% CI [0.199, 0.359]). The interval did not contain zero, demonstrating a statistically significant direct effect. The mediating effect size of self-efficacy was 0.152 (95% CI [0.103, 0.208]). The mediating effect size of perceived social support was 0.063 (95% CI [0.035, 0.094]). Neither interval included zero, indicating that the association between financial toxicity and exercise adherence was mediated by both self-efficacy and perceived social support.

**Table 3:** Results of the mediation effect analysis.

**Effect**	**β**	**Bootstrap 95% CI**		**Proportion of effect**
		**lower**	**upper**	
Direct effect: Financial toxicity → Exercise adherence	0.279	0.199	0.359	56.6%
Indirect effect 1: Financial toxicity → Self-efficacy → Exercise adherence	0.152	0.103	0.208	30.8%
Indirect effect 2: Financial toxicity → Perceived social support → Exercise adherence	0.063	0.035	0.094	12.8%
Total indirect effect	0.215	0.158	0.276	43.6%
Total effect	0.493	0.409	0.578	100%

## 4 Discussion

Based on stress and coping theory, this study constructed and tested a parallel mediation model between financial toxicity and exercise adherence among stroke patients. It examined the hypothesized pathways linking financial toxicity and exercise adherence and identified statistically significant indirect associations via self-efficacy and PSS.

In this study, stroke patients scored 20.57 ± 7.52 on the financial toxicity scale, indicating mild financial toxicity. This mean score was lower than that reported by Zhang et al. (38) among stroke patients. One possible explanation is that 48.6% of participants in the present study had moderate to severe dependence in activities of daily living. Such patients typically require more intensive healthcare resources, which increases medical expenditures and may heighten the risk of financial toxicity. Moreover, if work is interrupted due to diminished self-care abilities and reduced labor capacity, the risk of financial toxicity will be further exacerbated. Therefore, future studies should adopt multicenter designs to improve the generalizability of findings.

The present study showed a strong association between financial toxicity and exercise adherence among stroke patients. The direct effect of financial toxicity on exercise adherence was statistically significant (effect size = 0.279, 95% CI [0.199, 0.359]), representing 56.6% of the total effect. Patients with more severe financial toxicity tended to report lower levels of exercise adherence. This finding is consistent with Xu et al. (7), who found in a qualitative study that some stroke patients discontinued rehabilitation or delayed follow-up examinations due to financial pressures. Previous studies have also indicated that financial toxicity can adversely affect patients’ treatment adherence (5). One possible explanation is that patients experiencing financial toxicity may take steps to reduce their financial burden, which may affect behaviors related to adherence. Related studies also provide supportive evidence for this interpretation. A qualitative study of patients with chronic obstructive pulmonary disease found that financial toxicity may contribute to non-adherence to medical advice, discontinuation of medical care, or refusal of treatment. Some patients also sought cheaper alternative treatment options to reduce financial burden (39). Another study showed that financial toxicity could lead some patients to discontinue treatment because of concerns about debt and the financial burden on their families (40). The findings of this study provide evidence that financial toxicity is an important factor related to stroke patients’ health behaviors, including exercise adherence during rehabilitation.

The results also indicated that self-efficacy partially mediated the association between financial toxicity and exercise adherence among stroke patients. This suggests that financial toxicity may indirectly reduce exercise adherence by weakening patients’ rehabilitation self-efficacy. This is consistent with Sadigh et al. (41), who reported that patients with more severe financial toxicity tended to have lower levels of self-efficacy. Self-efficacy plays an important role in sustaining rehabilitation behaviors. Patients with higher levels of self-efficacy may cope with difficulties related to illness in positive ways. They may also be better able to regulate negative emotions such as anxiety, which may make it easier for them to maintain exercise behaviors (42, 43). The findings highlight the need to address psychological resources alongside financial stressors. Therefore, in clinical practice, multiple approaches can be employed to stimulate patients’ intrinsic motivation and enhance their self-efficacy.

The findings of this study also showed that perceived social support partially mediated the association between financial toxicity and rehabilitation exercise adherence among stroke patients. This suggests that financial toxicity may indirectly reduce exercise adherence by lowering patients’ perceived social support. One possible explanation is that, as financial toxicity increases, patients may perceive fewer supportive resources and may be less inclined to seek help because they worry about burdening others, which may be associated with lower perceived social support. Reduced perceived social support may further reduce exercise adherence. Consistent with this interpretation, previous research has reported a significant positive association between financial toxicity and social support, and suggested that social support can reduce stress related to financial toxicity (44). Studies further found that encouragement from family members, friends, and peers is an important factor influencing exercise adherence among stroke patients (45). When patients perceive insufficient social support, they may receive less emotional encouragement and practical assistance, thereby affecting their health behaviors (46). This indicates that in clinical practice, family members or significant others should be involved in developing rehabilitation plans and follow-up care, while promoting peer interaction to enhance patients’ perception of social support.

Notably, this study verified the parallel mediating roles of self-efficacy and perceived social support between financial toxicity and rehabilitation exercise adherence. The total indirect effect was 0.215 (95% CI [0.158, 0.276]), accounting for 43.6% of the total effect. Among the two mediating pathways, the indirect effect through self-efficacy was larger, with an effect size of 0.152 (95% CI [0.103, 0.208]), explaining 30.8% of the total effect. The indirect effect through perceived social support was 0.063 (95% CI [0.035, 0.094]), accounting for 12.8% of the total effect. This suggests that financial toxicity may influence patients’ rehabilitation behaviors through multiple pathways. From the perspective of stress and coping theory, self-efficacy serves as an individual’s internal psychological resource, while perceived social support functions as an external social resource. Both possess relatively independent mechanisms of action in coping with stressful events. Therefore, the findings suggest that clinical interventions should focus on both strengthening patients’ self-efficacy and enhancing social support resources. This may help reduce the negative effects of financial toxicity on rehabilitation exercise adherence.

## 5 Limitations

This study has certain limitations. First, this study used a cross-sectional design, and the links among variables only reflect statistical associations. Causal relationships cannot be inferred. The causal relationships among variables could be confirmed by future longitudinal studies. Second, participants were enrolled from only one tertiary hospital, which may restrict the generalizability of the results. Future studies could conduct multicenter research in different regions and healthcare settings to improve the representativeness and generalizability of the findings. Third, this study was designed as a quantitative study, and the pathways discussed were mainly interpreted based on statistical associations, the theoretical framework, and previous literature. Therefore, patients’ subjective feelings in the process by which financial toxicity influences exercise adherence could not be directly captured. In the future, qualitative research could be conducted to explore patients’ subjective feelings and lived experiences in this process.

## 6 Conclusion

The findings showed that financial toxicity was significantly correlated with exercise adherence among patients with stroke. The association between financial toxicity and exercise adherence was partially mediated by self-efficacy and perceived social support. To improve exercise adherence, attention should be paid not only to financial toxicity but also to enhancing patients’ self-efficacy and levels of social support.

## Data Availability

The raw data supporting the conclusions of this article will be made available by the authors, without undue reservation.

## Footnotes

1
https://schoemanna.shinyapps.io/mc_power_med/
